# Long non-coding RNA XIST as a potential prognostic biomarker in human cancers: a meta-analysis

**DOI:** 10.18632/oncotarget.23744

**Published:** 2017-12-26

**Authors:** Shaopu Hu, Junli Chang, Yimian Li, Wenyi Wang, Mengyao Guo, Edward C. Zou, Yongjun Wang, Yanping Yang

**Affiliations:** ^1^ Longhua Hospital, Shanghai University of Traditional Chinese Medicine, Shanghai, 200032, China; ^2^ School of Rehabilitation Science, Shanghai University of Traditional Chinese Medicine, Shanghai, 201203, China; ^3^ Key Laboratory of Theory and Therapy Of Muscles And Bones, Ministry of Education, Shanghai, 200032, China; ^4^ Consulting Engagement Management, Cerner, Kansas City, MO, 64117, USA

**Keywords:** lncRNA, XIST, human cancer, meta-analysis, prognostic biomarker

## Abstract

Growing studies have confirmed that long non-coding RNAs (lncRNAs) involve in the occurrence and development of various cancers. XIST, as a lncRNA, was dysregulated in different cancers. This meta-analysis was performed to evaluate the prognostic potential of XIST in malignant tumors. Eight databases of PubMed, Web of Science, Embase, Cochrane library, CNKI, VIP, SinoMed and Wang Fang were comprehensively searched from their initiation date to August 15, 2017. A total of nine studies with 853 cancer patients met the including criteria were finally included in this meta-analysis after independently screening the literatures by two researchers. Any discrepancies were resolved by a consensus. Hazard ratios (HRs) with corresponding 95% confidence intervals (CIs) for the primary endpoints were extracted and pooled for meta-analysis. Our results showed that expression level of XIST was markedly associated with overall survival (function as oncogene, HR = 0.53, 95% CI: 0.42–0.68, *p* < 0.00001; function as tumor suppressor, HR = 2.25, 95% CI: 1.15–4.37, *p* = 0.02), disease free survival (DFS)(HR = 0.45; 95% CI: 0.31–0.67, *p* < 0.0001), tumor type (digestive system carcinoma, HR = 0.50; 95% CI: 0.37–0.69, *p* < 0.00001; non-digestive system carcinoma, HR = 0.58; 95% CI: 0.39–0.87, *p* = 0.008), lymph node metastasis (OR = 0.32, 95% CI: 0.20–0.52, *p* < 0.00001), distant metastasis (OR = 0.36, 95% CI: 0.22–0.60, *p* < 0.0001) and tumor stage (OR = 0.43, 95% CI: 0.31–0.60, *p* < 0.00001). In conclusion, the pooled results in our current work suggest that XIST is an important prognostic biomarker in cancer patients.

## INTRODUCTION

With the development of deep sequencing methodologies, the function of non-coding RNAs (ncRNAs) have attracted a wide attention. The value of these so called “dark matter” was used to be underestimated immensely due to ncRNAs were considered not involved in the encoding proteins. However, ncRNAs actually participate in a variety of biological processes [1]. Long non-coding RNAs (lncRNAs) belong to ncRNA family with the length more than 200 nucleotides [2]. Accumulating reports indicated that lncRNAs were dysregulated in various types of cancers, including breast cancer [3, 4], colon cancer [5], hepatocellular carcinoma [6], gastric cancer [7, 8], osteosarcoma [9, 10] and so on. The dysregulation of lncRNAs is associated with tumor progression and the prognosis of cancer patients.

LncRNA X-inactive specific transcript (XIST), a product of the XIST gene, is the master regulator of X inactivation in mammals, and XIST gene is exclusively transcribed from the inactive X chromosome [11, 12]. Considerable evidence has shown that lncRNA XIST is dysregulated in a variety of human cancers, and this dysfunctional expression is associated with the clinicopathologic characteristics in cancer patients, including overall survival (OS), tumor stage, lymph node metastasis (LNM), and distant metastasis (DM) [13–15]. These discoveries indicate that XIST may be a potential prognostic biomarker for human cancers.

Therefore, we comprehensively searched relevant literatures to identify the associations between the abnormal expression of lncRNA XIST and clinicopathologic characteristics of cancer patients, and carried out this meta-analysis to evaluate whether lncRNA XIST could serve as a potential prognostic biomarker in cancer patients.

## RESULTS

### Literature identification and selection

**Figure 1:**
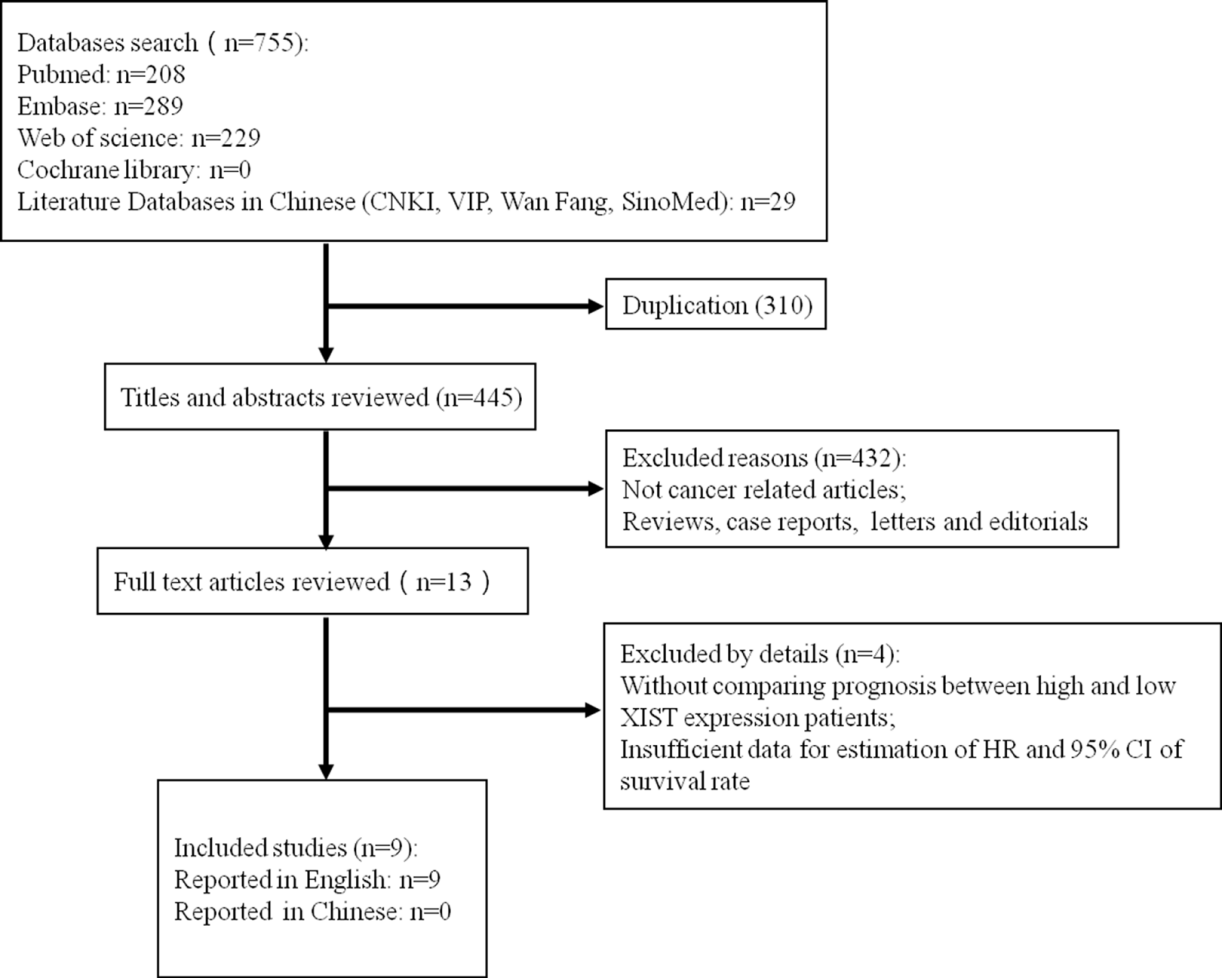
The flow diagram of process for the literature identification and selection

The detailed process of the literature identification and selection is shown in Figure 1. A total of 755 publications were retrieved according to the search strategy described in the section of methods, while 310 of the duplicated ones were excluded. After reviewing the titles and abstracts, 432 additional literatures were removed because of not cancer related articles or not original research works (Reviews, case reports, letters and editorials). After reading the full text of the 13 remaining publications, three of them were further excluded because of articles without comparing the prognosis between high and low XIST expression patients or providing insufficient data for estimation of HR and 95% CI for survival rate [11, 16, 17]. Among the remaining 10studies, only 1 study evaluated the association between XIST expression levels and the progression free survival (PFS) [18], therefore this study could not be pooled and included in this meta-analysis. Finally, nine of the literatures and 853 patients coincided with the inclusion criteria were included in this meta-analysis. All included publications were reported in English [13–15, 19–24].

### Study characteristics

The main characteristics of the nine studies are summarized in Table 1. Eight of the nine studies were from China and one study was from Japan. The number of patients enrolled in the nine included studies was between 49 and 145. Two of the nine studies focused on gastric cancer, two on hepatocellular carcinoma, each one on nasopharyngeal carcinoma, pancreatic cancer, osteosarcoma, esophageal squamous cell carcinoma or cervical squamous cell carcinoma. The diagnosis of LNM, DM and tumor stage was depended on the pathology. Patients in all articles were divided into high and low XIST expression groups. Seven of the nine studies reported the expression level of XIST was up-regulated in cancer tissues and cell lines, 2 studies reported it was down-regulated. The Newcastle-Ottawa Scale (NOS) scores of all included studies were ≥ 7.

**Table 1:** The characteristics of studies included in this meta-analysis

Study	Year	Region	Tumor type	Reference gene	Sample size	XIAT expression						HR (95% CI) Low/High	Outcome	Expression level	Method	NOS
						Low			High							
						Total	LNM	DM	Total	LNM	DM					
Dong-liang Chen [13]	2016	China	GC	GAPDH	106	52	31	8	54	44	20	0.41 (0.20–0.86)	OS	↑	qRT-PCR	8
Lei Ma [19]	2017	China	GC	GAPDH/U6	98	53	22	-	45	33	-	0.53 (0.30–0.91)	OS	↑	qRT-PCR	7
Peng Song [22]	2016	China	NPC	GAPDH/U6	108	32	-	-	76	-	-	0.58 (0.27–1.24)	OS	↑	qRT-PCR	8
Wei Wei [23]	2017	China	PC	RNU6B	64	32	11	11	32	17	18	0.44 (0.22–0.89)	OS	↑	qRT-PCR	7
G.-L. LI [15]	2017	China	OSC	GAPDH	145	70	-	14	75	-	30	0.59 (0.37–0.94)	OS	↑	qRT-PCR	8
Xiaoliang Wu [24]	2017	China	ESCC	GAPDH	127	63	-	-	64	-	-	OS:0.58 (0.34–1.01) DFS:0.51 (0.31–0.84)	OS/DFS	↑	qRT-PCR	8
Yichao Mo [21]	2017	China	HCC	GAPDH/U6	88	50	-	-	38	-	-	0.39 (0.21–0.69)	DFS	↑	qRT-PCR	7
Weijie Ma [20]	2017	China	HCC	GAPDH	68	38	-	-	30	-	-	2.53 (1.18–5.44)	OS	↓	qRT-PCR	8
Reiko Kobayashi [14]	2016	Japan	CSCC	GAPDH	49	25	6	-	24	11	-	1.54 (0.40–5.94)	OS	↓	RTqPCR	7

Abbreviations: GC, gastric cancer; HCC, hepatocellular carcinoma; NPC, nasopharyngeal carcinoma; PC, pancreatic cancer; OSC, osteosarcoma; ESCC, esophageal squamous cell carcinoma; CSCC, cervical squamous cell carcinoma; LNM, lymph node metastasis; DM, distant metastasis; OS, overall survival; DFS, disease free survival; HR: hazard ratios; CI: confidence intervals.

### Association between XIST expression levels and OS

**Figure 2:**
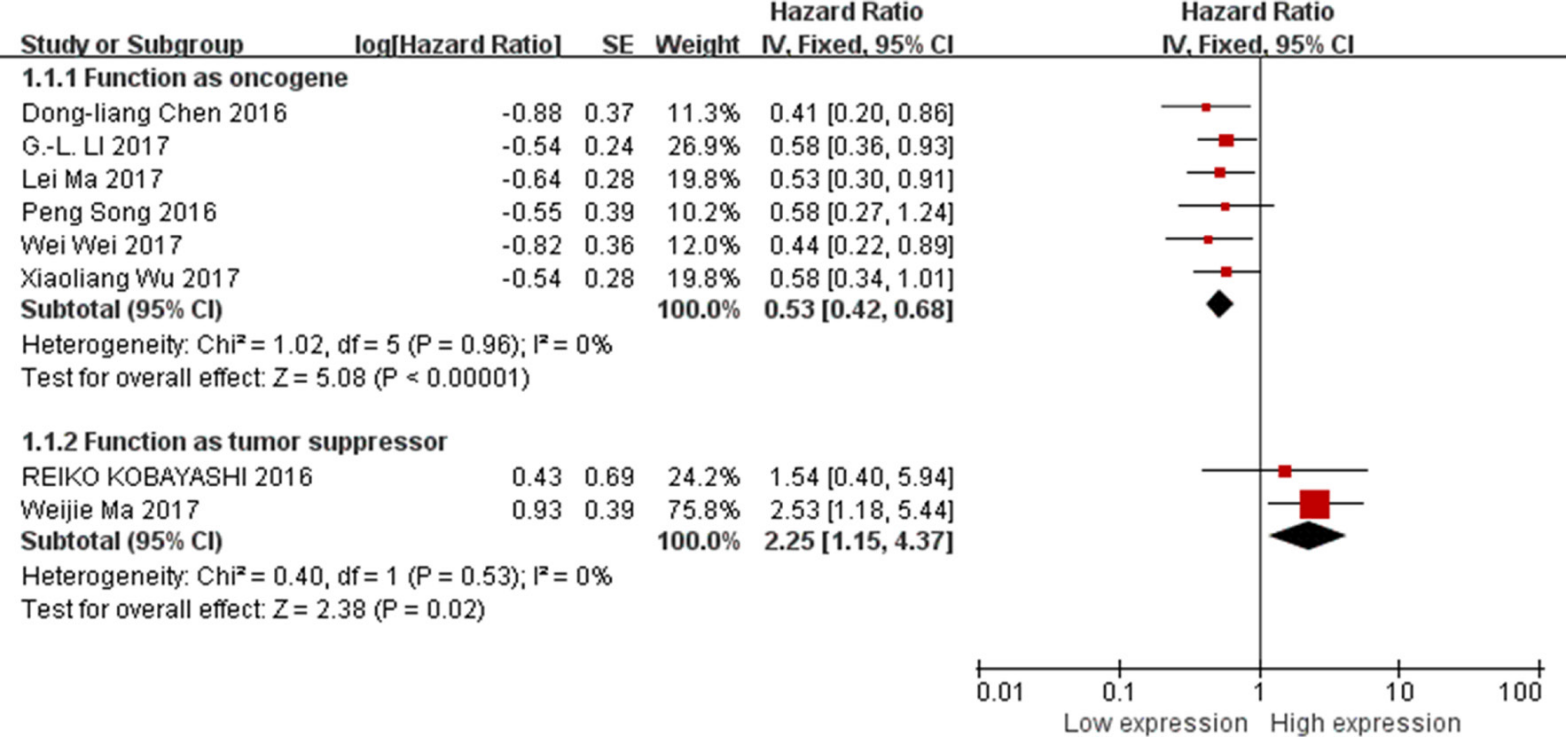
Forest plot for the association between XIST expression levels with OS

There were eight studies evaluated the association between XIST expression levels and OS in this meta-analysis, and six of them reported XIST function as the oncogenes. Therefore, all the data from these six studies were extracted and pooled for reanalysis [13, 15, 19, 22–24]. There was no any significant heterogeneity among the studies (I^2^ = 0%), therefore, fixed-effects model was used. The results of the forest plot suggested that XIST was associated with OS of cancer patients (pooled HR = 0.53, 95% CI: 0.42–0.68, *p* < 0.00001; Figure 2, upper part). Therefore, our data here indicated that higher XIST expression in the tumor tissues of patients with gastric cancer, nasopharyngeal carcinoma, pancreatic cancer, osteosarcoma or esophageal squamous cell carcinoma were associated with a shorter OS.

Data from other 2 studies reported XIST functions as the tumor suppressors were collected and reanalyzed [14, 20]. The results of the forest plot indicated that lower expression of XIST in the tumor tissues of patients with hepatocellular carcinoma or cervical squamous cell carcinoma was associated with shorter OS. The pooled HR = 2.25; 95% CI: 1.15–4.37, *p* = 0.02 (Figure 2, lower part).

### Association between XIST expression levels and tumor types

**Figure 3:**
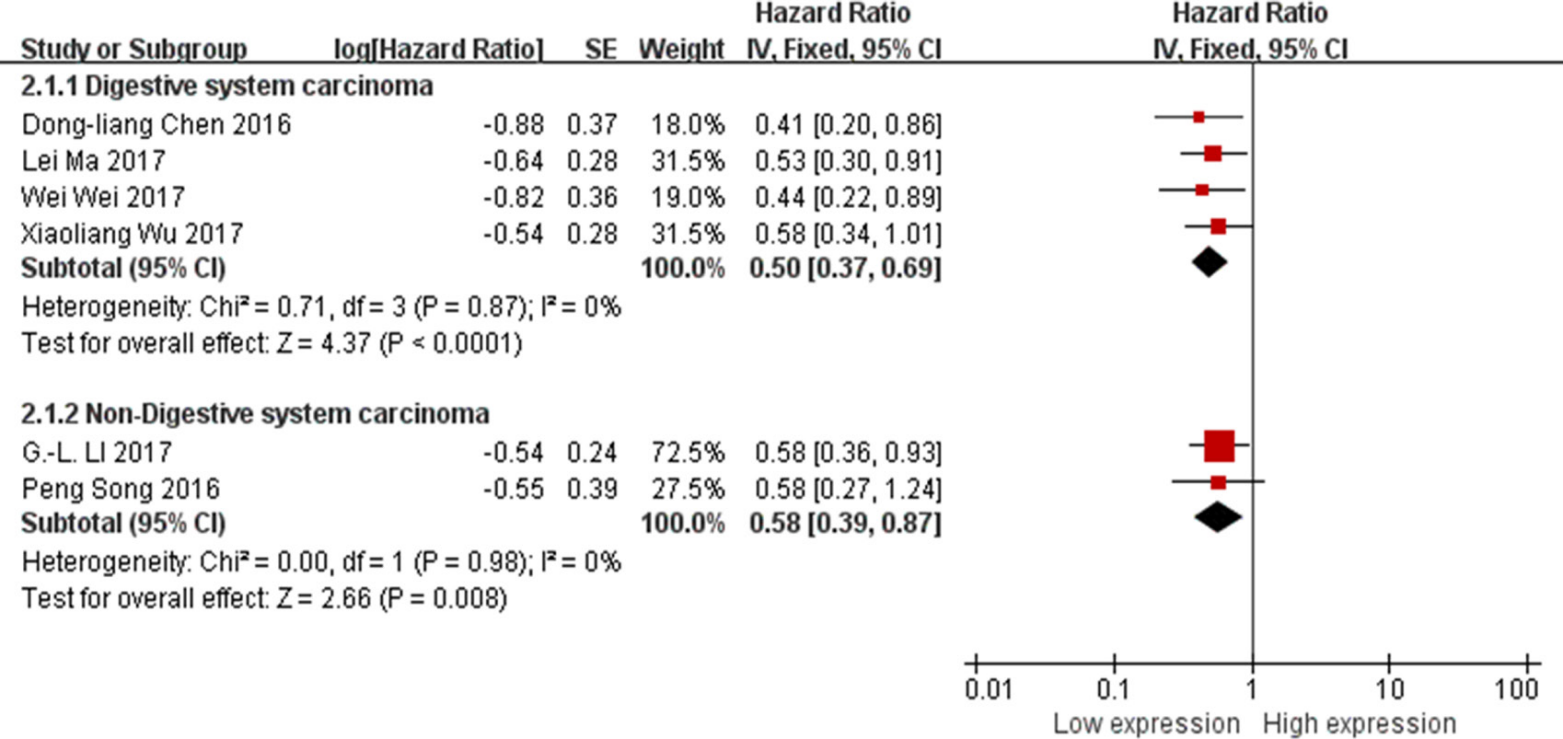
Forest plot for the association between XIST expression levels with tumor types

Among all 6 studies, reported XIST as the oncogene and evaluated the association between XIST expression levels and OS, 4 studies [13, 19, 23, 24] explored the digestive system carcinoma and other 2 studies [15, 22] explored the non-digestive system carcinoma. Therefore, all the data from these 6 studies were extracted and pooled for reanalysis according to the digestive system or non-digestive system tumor. Fixed-effects model was used due to no any heterogeneity among the studies (I^2^ = 0%). The results of the forest plot suggested that among cancer patients with XIST as the oncogenes, digestive system carcinoma patients with gastric cancer, pancreatic cancer or esophageal squamous cell carcinoma showed a shorter OS than non-digestive system carcinoma patients with nasopharyngeal carcinoma or osteosarcoma (digestive system carcinoma, HR = 0.50; 95% CI: 0.37–0.69, *p* < 0.0001; non-digestive system carcinoma, HR = 0.58; 95% CI: 0.39–0.87, *p* = 0.008. Figure 3).

There were 2 studies that reported XIST as the tumor suppressor and evaluated the association between XIST expression levels and OS. One study reported that XIST was down-regulated in hepatocellular carcinoma tissues, and patients with lower XIST expression showed shorter OS rates than those with higher XIST expression [20]; the other study reported that XIST was down-regulated in cervical squamous cell carcinoma tissues, and higher XIST expression showed good prognosis than lower XIST expression [14], therefore, the data from these 2 studies could not be pooled.

### Association between XIST expression levels and disease free survival

**Figure 4:**
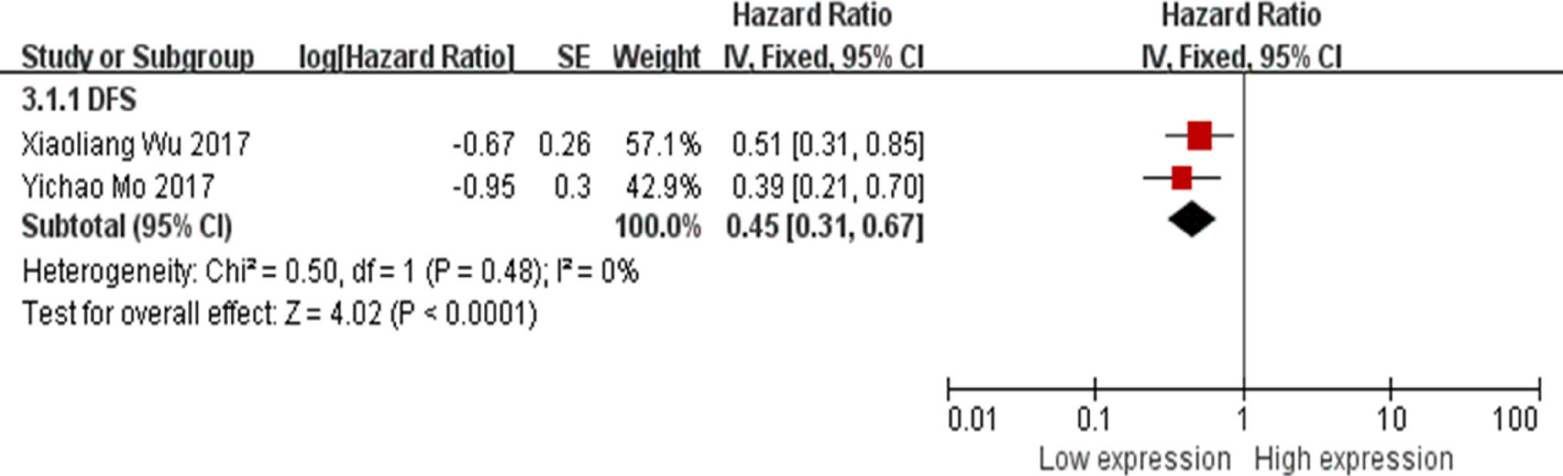
Forest plot for the association between XIST expression levels with DFS

There were 2 studies evaluated the association between XIST expression levels and disease free survival (DFS) [21, 24]. Therefore, all the data were extracted from the 2 studies and pooled for reanalysis. The fixed-effects model was applied because of no any significant heterogeneity among the studies (I^2^ = 0%). The results of the forest plot demonstrated that hepatocellular carcinoma or esophageal squamous cell carcinoma patients with higher expression of XIST in the tumor tissues were associated with shorter DFS. The pooled HR = 0.45; 95% CI: 0.31–0.67, *p* < 0.0001 (Figure 4).

### Association between XIST expression levels and LNM

**Figure 5:**
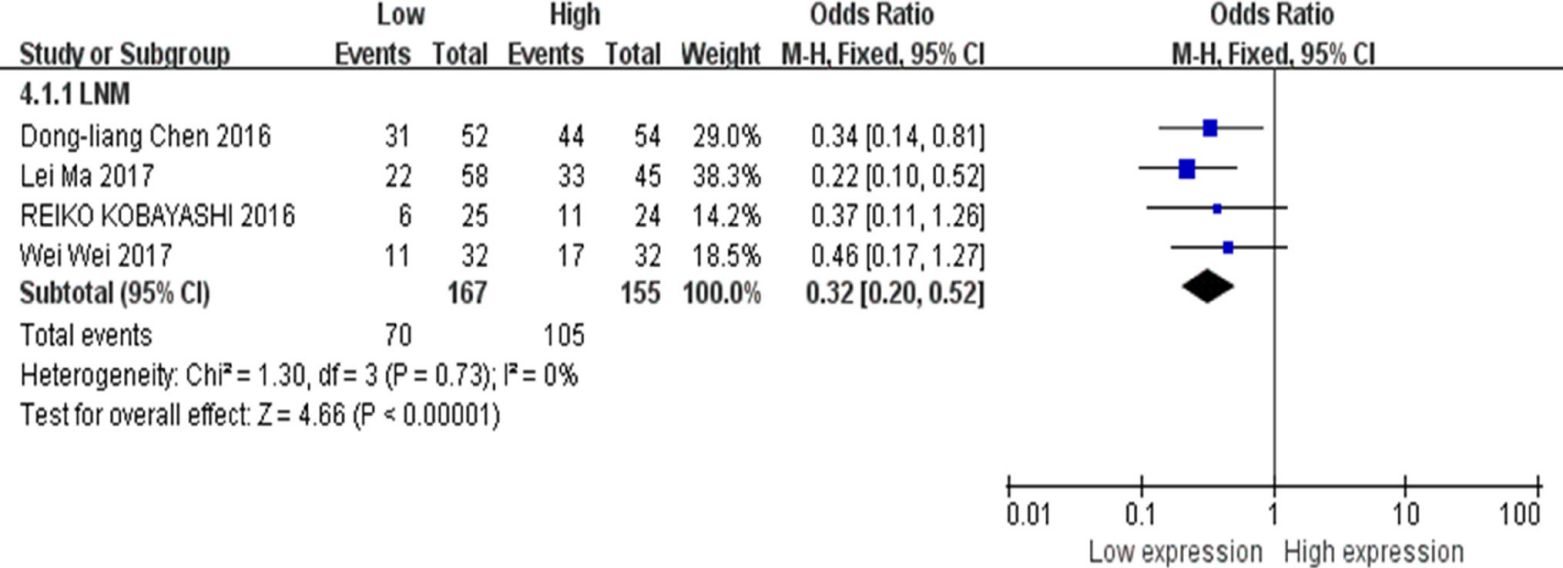
Forest plot for the association between XIST expression levels with LNM

There were 4 studies evaluated the association between XIST expression levels and LNM [13, 14, 19, 23], thus data of 322 patients were collected and reanalyzed. The fixed-effects model was applied because of no any significant heterogeneity among the studies (I^2^ = 0%). The results of the forest plot demonstrated that gastric cancer, pancreatic cancer or cervical squamous cell carcinoma patients with higher expression of XIST in the tumor tissues, were more liable to developing LNM. The pooled OR = 0.32; 95% CI: 0.20–0.52, *p* < 0.00001 (Figure 5).

### Association between XIST expression levels and DM

**Figure 6:**
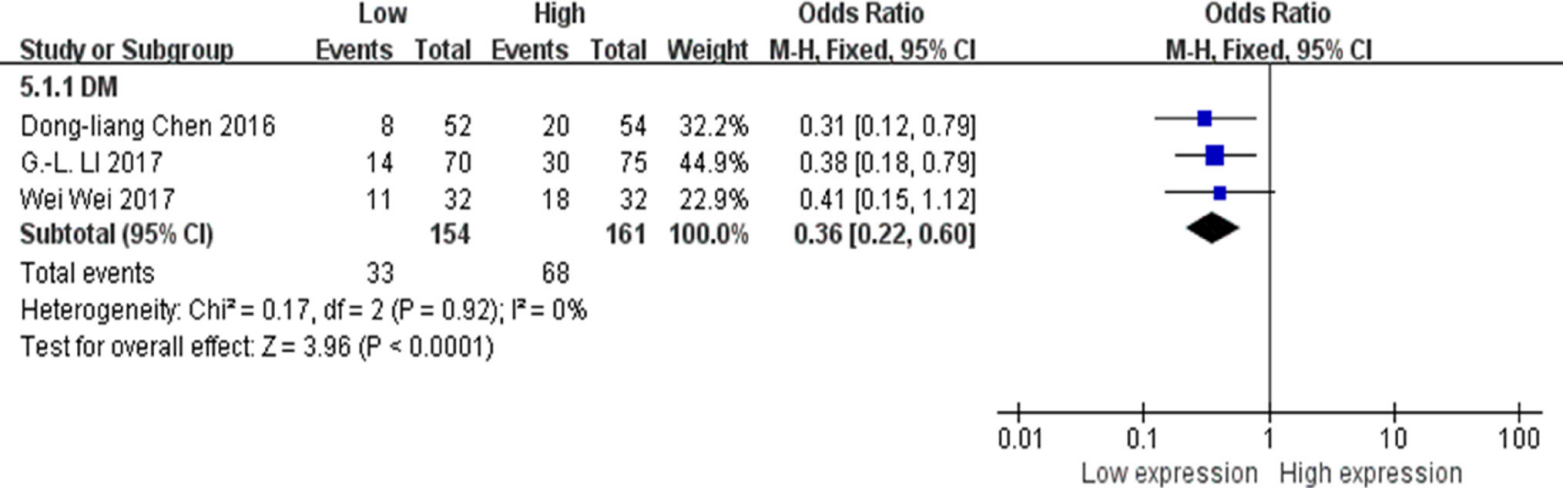
Forest plot for the association between XIST expression levels with DM

There were 3 studies evaluated the association between XIST expression levels and DM [13, 15, 23], data of 315 patients were collected and reanalyzed. The fixed-effects model was adopted because of no any significant heterogeneity among these studies (I^2^ = 0%). The results of the forest plot demonstrated that gastric cancer, pancreatic cancer or osteosarcoma patients with higher expression of XIST in the tumor tissues were more prone to DM. The pooled OR = 0.36; 95% CI: 0.22–0.60, *p* < 0.0001 (Figure 6).

### Association between XIST expression levels and tumor stages

**Figure 7:**
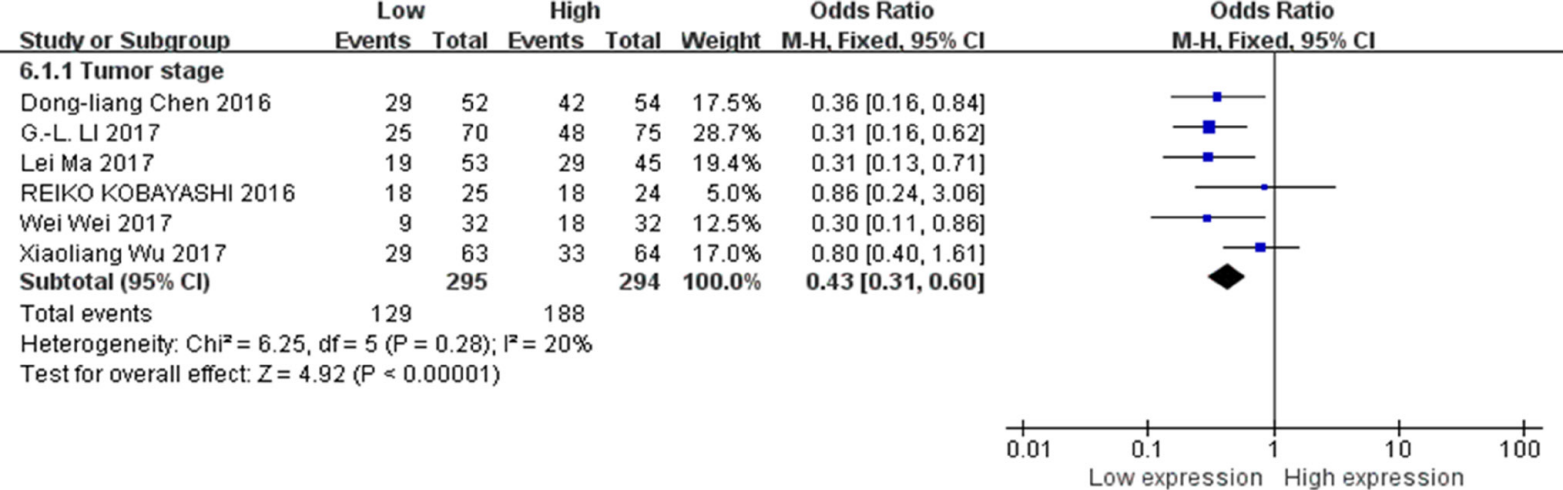
Forest plot for the association between XIST expression levels with tumor stages

There were 6 studies reported the association between XIST expression levels and tumor stages [13–15, 19, 23, 24]. Data of 589 patients were collected and analyzed. The fixed-effects model was adopted because of no significant heterogeneity among the studies (I^2^ = 20%). The results of the forest plot demonstrated that gastric cancer, pancreatic cancer, osteosarcoma, esophageal squamous cell carcinoma or cervical squamous cell carcinoma patients with higher expression of XIST in the tumor tissues may have increased probability of high tumor stages. The pooled OR = 0.43; 95% CI: 0.31–0.60, *p* < 0.00001 (Figure 7).

## DISCUSSION

In the recent years, researchers tend to explore the potential value of lncRNAs in human diseases, especially in cancer. Accumulating evidences indicated that expressions of some lncRNAs were dysregulated in various cancers, and lncRNAs can act as oncogenic or tumor suppressor roles based on these expression levels in tumor tissues or cells. For example, lncRNA HULC expression was up-regulated in osteosarcoma tissues and cell lines, and the higher expression of HULC was associated with poor prognosis of osteosarcoma patients. Osteosarcoma cell proliferation, migration and invasion were inhibited by suppressing HULC expression [25]. LncRNA UCA1 was up-regulated in esophageal squamous cell carcinoma, gastric cancer tissues and cell lines, knockdown of UCA1 could inhibit proliferation of cancer cells [26].

The number of published literatures to explore the association between lncRNAs and prognosis of cancer patients is enormous. The reliability of the experimental conclusions is often affected by the sample size. Therefore, comprehensive searching the relevant studies and performing a meta-analysis can get a reliable conclusion. For example, two published meta-analyses individually analyzed the possibility of lncRNA HULC [27] or UCA1 [28] as a potential prognostic biomarker in the human cancer. The reanalysis of a pooled large sample size provides a basis for future clinical and experimental design.

LncRNA XIST has been found to be dysregulated in several cancers, and associated with aggressive tumor phenotypes and poor prognosis in cancer patients. XIST is associated with tumor progression through targeting miR-34a-5p or miR-497/MACC1 axis in nasopharyngeal carcinoma [22] and gastric cancer [19] individually. Moreover, XIST mediated oncogenic effects is partially through its epigenetically silencing of KLF2 expression in non-small cell lung cancer [18]. Meanwhile, XIST has been recently found to be down-regulated in hepatocellular carcinoma [21] and cervical squamous cell carcinoma [24], and associated with the prognosis of these cancer patients.

Our current meta-analysis evaluated the association between XIST expression levels and tumor types, LNM, DM, tumor stages, DFS and OS. Our data indicated that abnormal expression of XIST was significant correlated with OS (function as oncogene, HR = 0.53, 95% CI: 0.42–0.68, *p* < 0.00001; function as tumor suppressor, HR = 2.25, 95% CI: 1.15–4.37, *p* = 0.02), DFS (HR = 0.45; 95% CI: 0.31–0.67, *p* < 0.0001) tumor types (digestive system carcinoma, HR = 0.50; 95% CI: 0.37–0.69, *p* < 0.0001; non-digestive system carcinoma, HR = 0.58; 95% CI: 0.39–0.87, *p* = 0.008), LNM (OR = 0.32, 95% CI: 0.20–0.52, *p* < 0.00001), DM (OR = 0.36, 95% CI: 0.22–0.60, *p* < 0.0001) and the tumor stages (OR = 0.43, 95% CI: 0.31–0.60, *p* < 0.00001). Based on these results, we concluded that XIST might serve as a prognostic biomarker in human cancers of gastric cancer, hepatocellular carcinoma, nasopharyngeal carcinoma, pancreatic cancer, osteosarcoma, esophageal squamous cell carcinoma and cervical squamous cell carcinoma.

Among all included studies in this meta-analysis, two studies evaluated the expression level of lncRNA XIST and patient prognosis in hepatocellular carcinoma. One study [20] reported that XIST expression was significantly down-regulated in hepatocellular carcinoma specimens in comparison with the adjacent normal tissues, while another study [21] demonstrated XIST was upregulated in HCC tissues and cell lines. Two different results indicate that the abnormal expression level of XIST can be affected by different experimental conditions or tumor tissues. Inconsistent experimental results can enrich our understanding of the mechanism of XIST in hepatocellular carcinoma, while rigorous experimental design and result repetition are required in the future experiments.

Nevertheless, several limitations in this meta-analysis should be emphasized. First, among all included studies, eight studies were came from China, only one study was from Japan, therefore, the results of our data are not a global representative. Second, only nine studies included in this meta-analysis, the reliability of the results may be over-concluded. Therefore, larger-size and better designed studies are necessary to be conducted to confirm our conclusion.

In summary, our results suggest that abnormal XIST expression in human cancer is associated with OS and DFS of patients. Among cancer patients with XIST as the oncogenes, digestive system carcinoma patients were more closely associated with shorter OS than non-digestive system carcinoma patients. Moreover, patients with higher XIST expression in the tumor tissues were prone to developing LNM, DM, as well as increased probability of high tumor stages. These findings demonstrated that XIST could serve as a potential prognostic biomarker in human cancers of gastric cancer, hepatocellular carcinoma, nasopharyngeal carcinoma, pancreatic cancer, osteosarcoma, esophageal squamous cell carcinoma and cervical squamous cell carcinoma.

## MATERIALS AND METHODS

### Literature search strategy

PubMed, Web of Science, Embase, Cochrane library, CNKI, VIP, SinoMed and Wang Fang Database from their initiation date to August 15, 2017 were searched by two investigators (Shaopu Hu and Junli Chang). All covered literatures that evaluated the association between XIST expression levels and prognosis of cancer patients, without language limitation, were collected. The terms of (((((carcinoma) OR neoplasm) OR tumor) OR cancer)) AND (((long non coding RNA XIST) OR XIST) OR lncRNA XIST) were used as the search strategy.

### Inclusion and exclusion criteria

Inclusion criteria are as the following: 1) studies evaluating the expression level of XIST and patient prognosis in cancers; 2) patients were divided into low XIST expression group and high XIST expression group; 3) data of lymph node metastasis, distant metastasis, tumor stage, overall survival, disease free survival or progression free survival were reported; 4) method of measuring XIST expression level was reported; 5) hazard ratios (HR) and 95% confidence interval (CI) were described or could be indirectly calculated according to the survival curves.

Exclusion criteria are as the following: 1) studies without evaluation of XIST expression level and tumor prognosis; 2) non-clinical study, letters, expert opinions, reviews and case reports; 3) necessary data can not be extracted.

### Data extraction and quality assessment

The data of included studies were extracted independently by 3 reviewers (Yimian Li, Wenyi Wang and Edward C. Zou). If there were any divergences, a discussion was performed with another investigator (Yanping Yang). The following information were extracted from each included study: first author, publication year, country, tumor type, reference gene, sample size, total case in high XIST expression group and low XIST expression group, number of patients with LNM and DM in each group, detection methods, HRs and 95% CIs for OS or DFS.

The Newcastle-Ottawa Scale (NOS) [29] was applied to assess the quality of all included studies by two investigators (Mengyao Guo and Yongjun Wang) independently. The total scores for different studies ranged from 0 to 9. The study was considered to be high quality if the score was ≥ 7 (Table 2). All included studies of this meta-analysis were assessed to be of high quality.

**Table 2:** Methodological quality of included studies

Newcastle–Ottawa Scale (NOS) Quality Assessment Table				
Study	Selection	Comparability	Exposure/Outcome	Total Star
Dong-liang Chen [13]	++++	+	+++	8
Lei Ma [19]	++++	+	++	7
Peng Song [22]	++++	+	+++	8
Wei Wei [23]	+++	+	+++	7
G.-L. LI [15]	++++	+	+++	8
Xiaoliang Wu [24]	++++	+	+++	8
Yichao Mo [21]	++++	+	++	7
Weijie Ma [20]	++++	+	+++	8
Reiko Kobayashi [14]	++++	+	++	7

A star system was used to allow a semiquantitative assessment of study quality. A study could be awarded a maximum of 1 star for each numbered item within the selection and exposure categories. A maximum of 2 stars could be given for comparability. The NOS ranged from 0 to 9 stars. Studies achieved 7 were considered as high-quality ones, 4 to 6 stars were medium-quality studies, and < 4 stars were poor-quality studies.

### Statistical analysis

All the statistical analyses in this meta-analysis were performed by REVIEW MANAGER 5.3 software, as recommended by the Cochrane Collaboration. Based on the reported Kaplan-Meier curve in each included studies, the HRs and 95% CIs were estimated using the software of Engauge Digitizer (version 4.1), according to the specific calculation methods reported in the literature [30]. The survival results were calculated by log HR and standard error (SE) values.

Moreover, the association between XIST expression levels and the tumor parameters (LNM, DM and tumor stage) were evaluated by ORs and 95% CIs. The heterogeneity of the eligible studies was evaluated by the *Q* and I^2^ test. The application of effects model depends on the heterogeneity among the studies. If no significant heterogeneity (I^2^ < 50%, *p* > 0.1) in the included studies, fixed-effects model was used to analyze the results; while random-effects model was applied for meta-analysis if significant heterogeneity (I^2^ ≥ 50%, *P* ≤ 0.1) existed in the eligible studies [27].

## References

[1] Ponjavic J, Ponting CP, Lunter G (2007). Functionality or transcriptional noise? Evidence for selection within long noncoding RNAs. Genome Res.

[2] Barra J, Leucci E (2017). Probing Long Non-coding RNA-Protein Interactions. Front Mol Biosci.

[3] Li W, Jia G, Qu Y, Du Q, Liu B, Liu B (2017). Long Non-Coding RNA (LncRNA) HOXA11-AS Promotes Breast Cancer Invasion and Metastasis by Regulating Epithelial-Mesenchymal Transition. Med Sci Monit.

[4] Luan T, Zhang X, Wang S, Song Y, Zhou S, Lin J, An W, Yuan W, Yang Y, Cai H, Zhang Q, Wang L (2017). Long non-coding RNA MIAT promotes breast cancer progression and functions as ceRNA to regulate DUSP7 expression by sponging miR-155-5p. Oncotarget.

[5] Chen SW, Zhu J, Ma J, Zhang JL, Zuo S, Chen GW, Wang X, Pan YS, Liu YC, Wang PY (2017). Overexpression of long non-coding RNA H19 is associated with unfavorable prognosis in patients with colorectal cancer and increased proliferation and migration in colon cancer cells. Oncol Lett.

[6] Wu L, Zhang L, Zheng S (2017). Role of the long non-coding RNA HOTAIR in hepatocellular carcinoma. Oncol Lett.

[7] Chen Y, Wei G, Xia H, Yu H, Tang Q, Bi F (2017). Down regulation of lincRNA-p21 contributes to gastric cancer development through Hippo-independent activation of YAP. Oncotarget.

[8] Li C, Liang G, Yang S, Sui J, Yao W, Shen X, Zhang Y, Peng H, Hong W, Xu S, Wu W, Ye Y, Zhang Z (2017). Dysregulated lncRNA-UCA1 contributes to the progression of gastric cancer through regulation of the PI3K-Akt-mTOR signaling pathway. Oncotarget.

[9] Wang B, Su Y, Yang Q, Lv D, Zhang W, Tang K, Wang H, Zhang R, Liu Y (2015). Overexpression of Long Non-Coding RNA HOTAIR Promotes Tumor Growth and Metastasis in Human Osteosarcoma. Mol Cells.

[10] Xie CH, Cao YM, Huang Y, Shi QW, Guo JH, Fan ZW, Li JG, Chen BW, Wu BY (2016). Long non-coding RNA TUG1 contributes to tumorigenesis of human osteosarcoma by sponging miR-9-5p and regulating POU2F1 expression. Tumour Biol.

[11] Tantai J, Hu D, Yang Y, Geng J (2015). Combined identification of long non-coding RNA XIST and HIF1A-AS1 in serum as an effective screening for non-small cell lung cancer. Int J Clin Exp Pathol.

[12] Yao Y, Ma J, Xue Y, Wang P, Li Z, Liu J, Chen L, Xi Z, Teng H, Wang Z, Li Z, Liu Y (2015). Knockdown of long non-coding RNA XIST exerts tumor-suppressive functions in human glioblastoma stem cells by up-regulating miR-152. Cancer Lett.

[13] Chen DL, Ju HQ, Lu YX, Chen LZ, Zeng ZL, Zhang DS, Luo HY, Wang F, Qiu MZ, Wang DS, Xu DZ, Zhou ZW, Pelicano H (2016). Long non-coding RNA XIST regulates gastric cancer progression by acting as a molecular sponge of miR-101 to modulate EZH2 expression. J Exp Clin Cancer Res.

[14] Kobayashi R, Miyagawa R, Yamashita H, Morikawa T, Okuma K, Fukayama M, Ohtomo K, Nakagawa K (2016). Increased expression of long non-coding RNA XIST predicts favorable prognosis of cervical squamous cell carcinoma subsequent to definitive chemoradiation therapy. Oncol Lett.

[15] Li GL, Wu YX, Li YM, Li J (2017). High expression of long non-coding RNA XIST in osteosarcoma is associated with cell proliferation and poor prognosis. Eur Rev Med Pharmacol Sci.

[16] Schouten PC, Vollebergh MA, Opdam M, Jonkers M, Loden M, Wesseling J, Hauptmann M, Linn SC (2016). High XIST and Low 53BP1 Expression Predict Poor Outcome after High-Dose Alkylating Chemotherapy in Patients with a BRCA1-like Breast Cancer. Mol Cancer Ther.

[17] Wang Z, Yuan J, Li L, Yang Y, Xu X, Wang Y (2017). Long non-coding RNA XIST exerts oncogenic functions in human glioma by targeting miR-137. Am J Transl Res.

[18] Fang J, Sun CC, Gong C (2016). Long noncoding RNA XIST acts as an oncogene in non-small cell lung cancer by epigenetically repressing KLF2 expression. Biochem Biophys Res Commun.

[19] Ma L, Zhou Y, Luo X, Gao H, Deng X, Jiang Y (2017). Long non-coding RNA XIST promotes cell growth and invasion through regulating miR-497/MACC1 axis in gastric cancer. Oncotarget.

[20] Ma W, Wang H, Jing W, Zhou F, Chang L, Hong Z, Liu H, Liu Z, Yuan Y (2017). Downregulation of long non-coding RNAs JPX and XIST is associated with the prognosis of hepatocellular carcinoma. Clin Res Hepatol Gastroenterol.

[21] Mo Y, Lu Y, Wang P, Huang S, He L, Li D, Li F, Huang J, Lin X, Li X, Che S, Chen Q (2017). Long non-coding RNA XIST promotes cell growth by regulating miR-139-5p/PDK1/AKT axis in hepatocellular carcinoma. Tumour Biol.

[22] Song P, Ye LF, Zhang C, Peng T, Zhou XH (2016). Long non-coding RNA XIST exerts oncogenic functions in human nasopharyngeal carcinoma by targeting miR-34a-5p. Gene.

[23] Wei W, Liu Y, Lu Y, Yang B, Tang L (2017). LncRNA XIST Promotes Pancreatic Cancer Proliferation Through miR-133a/EGFR. J Cell Biochem.

[24] Wu X, Dinglin X, Wang X, Luo W, Shen Q, Li Y, Gu L, Zhou Q, Zhu H, Li Y, Tan C, Yang X, Zhang Z (2017). Long noncoding RNA XIST promotes malignancies of esophageal squamous cell carcinoma via regulation of miR-101/EZH2. Oncotarget.

[25] Sun XH, Yang LB, Geng XL, Wang R, Zhang ZC (2015). Increased expression of lncRNA HULC indicates a poor prognosis and promotes cell metastasis in osteosarcoma. Int J Clin Exp Pathol.

[26] Li JY, Ma X, Zhang CB (2014). Overexpression of long non-coding RNA UCA1 predicts a poor prognosis in patients with esophageal squamous cell carcinoma. Int J Clin Exp Pathol.

[27] Fan YH, Wu MJ, Jiang Y, Ye M, Lu SG, Wu L, Zhu XG (2017). Long non-coding RNA HULC as a potential prognostic biomarker in human cancers: a meta-analysis. Oncotarget.

[28] He A, Hu R, Chen Z, Liao X, Li J, Wang D, Lv Z, Liu Y, Wang F, Mei H (2017). Role of long noncoding RNA UCA1 as a common molecular marker for lymph node metastasis and prognosis in various cancers: a meta-analysis. Oncotarget.

[29] Wells GA, Shea B, O’Connell D, Peterson J, Welch V, Losos M, Tugwell P (2000). The Newcastle–Ottawa Scale (NOS) for Assessing the Quality of Non-Randomized Studies in Meta-Analysis.

[30] Tierney JF, Stewart LA, Ghersi D, Burdett S, Sydes MR (2007). Practical methods for incorporating summary time-to-event data into meta-analysis. Trials.

